# Characterising Challenging Behaviour Following Stroke: A Retrospective Chart Review of Presentation, Management, and Outcomes

**DOI:** 10.3390/nursrep16020053

**Published:** 2026-02-03

**Authors:** Scott Lamont, Catherine E. Lightbody, Malabika Ghosh, Rebecca Jefferson, Ting-Li Su, Caroline L. Watkins

**Affiliations:** Applied Health Research Hub, University of Lancashire, Preston PR1 2HE, UK; celightbody@lancashire.ac.uk (C.E.L.); mghosh1@lancashire.ac.uk (M.G.); rljefferson1@lancashire.ac.uk (R.J.); tsu@lancashire.ac.uk (T.-L.S.); clwatkins@lancashire.ac.uk (C.L.W.)

**Keywords:** stroke care, stroke recovery, challenging behaviour, agitation, confusion, clinical documentation, care planning, care outcomes

## Abstract

**Background/Objectives:** Challenging behaviour post-stroke can complicate care and disrupt rehabilitation, yet its presentation and management are underreported. This study explored how such behaviours were documented in clinical records and managed in stroke settings, and how care delivery and discharge outcomes were documented in this context. **Methods:** A retrospective chart review was conducted across two NHS stroke units, covering all admissions between March and April 2022. Patient records were reviewed to capture demographic, clinical, and behavioural information, together with details relating to management and discharge. Challenging behaviour was identified retrospectively from clinical documentation during routine care. Descriptive statistics were used to summarise the data. **Results:** Forty-eight stroke admissions were examined, with challenging behaviour documented in thirteen patients (27%). Eleven had ischaemic stroke, with moderate severity common (n = 6), while inattention/neglect (n = 5) and infection (n = 4) were also documented. Behaviours were commonly recorded as confusion or agitation, and predominantly by nursing staff. Challenges to care delivery were documented in six of these patients, and additional professional input was provided for seven. Discharge delays were documented in patients with challenging behaviour, and the median length of stay was notably longer for this group (19 days compared with 7). Documentation of cognitive or delirium screening was uncommon. **Conclusions:** Challenging behaviour was documented in over a quarter of acute stroke admissions and was documented alongside greater care complexity and longer hospital stays. These preliminary descriptive findings from a small sample indicate a need for further exploration to better characterise challenging behaviour following stroke and its implications for care.

## 1. Introduction

Stroke is a leading cause of death and disability in the UK, with approximately 100,000 cases annually [1]. A substantial proportion of stroke survivors experience long-term physical, cognitive, emotional or behavioural deficits [2]. Although the term ‘challenging behaviour’ is not used explicitly in the stroke literature, a range of behavioural presentations are described that can challenge care delivery, including anger, aggression, irritability and agitation [3]. Confusion is also commonly documented after stroke and can present significant challenges for care [4]. Challenging behaviours in healthcare settings vary in intensity, frequency, and duration. They are recognised as a source of safety risks and care disruption, and they place emotional and physical demands on staff while also affecting patient experience [5,6]. An organisational imperative therefore exists in understanding challenging behaviours post-stroke, in mitigating their deleterious effects and maintaining ‘safety for all’.

Long recognised as significant post-stroke sequelae [7], challenging behaviours may appear at any stage of recovery and are often missed, particularly when communication or cognitive difficulties limit assessment [8,9]. Their presence, particularly agitation and aggression, can interfere with rehabilitation and day-to-day ward routines, impacting the delivery of care and overall recovery [10]. Nursing staff, at the forefront of managing challenging behaviour, frequently report heightened stress and increased emotional burden when managing such behaviours in neurorehabilitation settings [11,12]. Significant patient distress [13] and increased healthcare utilisation [14] have also been reported following stroke in relation to neuropsychiatric complications. These observations contribute to recommendations for strengthening general psychological support within stroke services [15] and highlight wider service impacts [16]. They also highlight the need for a comprehensive understanding of challenging behaviour following stroke.

A study exploring anger, hostility and aggression in acute stroke found 35% of patients presenting with anger [17]. The study highlighted that anger was probably caused by the stroke lesion which interfered with emotional control and noted that many patients were not aware of this presentation. Where post-stroke mania is present, agitation may be particularly prominent, with a systematic review reporting agitation in approximately 63% of the 74 reported cases [18]. Post-stroke anger (PSA) and aggression are among the most commonly reported behavioural symptoms, with prevalence estimates ranging from 11 to 35% in the acute phase and from 19 to 32% in the subacute phase [9]. There is a lack of consensus regarding the precipitating factors of PSA. Recent reports suggest a correlation with damage to brain regions responsible for emotional regulation [19]. Other studies suggest psychosocial stressors, such as frustration with functional limitations, as important contributing factors to post-stroke emotional and behavioural difficulties [20,21].

Previously, Kim [22] described ‘anger proneness’, a difficulty in regulating post-stroke aggression, and identified it, along with emotional incontinence, as factors negatively influencing functional outcomes and increasing carer burden. While the impact of this seems to be less severe than other emotional disturbances (e.g., post-stroke depression), it can be distressing and embarrassing for patients, decrease their quality of life [10] and negatively influence functional outcomes [22]. Despite their prevalence and impact, these behaviours remain insufficiently characterised in the literature, particularly as to how stroke services document and manage these behaviours in practice.

This study aimed to explore the presentation of challenging behaviour following acute stroke in relation to demographic and clinical characteristics, and to describe its documentation in relation to care and discharge.

## 2. Materials and Methods

Study Design

A retrospective chart review was conducted to explore the presentation, assessment, and management of challenging behaviour in patients admitted with acute stroke. The study is reported in accordance with the SQUIRE 2.0 (Standards for Quality Improvement Reporting Excellence) guidelines [23] and is available in the Supplementary Materials.

Context and Setting

The study was undertaken at two stroke units within the Lancashire Teaching Hospitals NHS Foundation Trust. These units provide acute stroke care and rehabilitation services, with multidisciplinary staffing including stroke physicians, nurses, occupational therapists, physiotherapists, and speech and language therapists. The study was undertaken as part of a 12-month academically supervised research internship, which included supervisor allocation, project scoping, data collection and analysis, report writing, and poster presentation. The research intern was a senior occupational therapist experienced in clinical practice. All patients consecutively admitted to the stroke units between March and April 2022 were included.

Data Collection

Electronic records were reviewed by a member of the study team (Principal Occupational Therapist). A data collection tool was developed to capture data across four domains: demographics (e.g., age, gender, pre-admission living arrangements); clinical history (e.g., stroke type, pre-existing confusion or psychiatric history, NIHSS score); documented impact on care (e.g., own care, other patients, additional staffing, management); and discharge and follow-up outcomes (e.g., discharge destination, delays, and post-discharge care). Challenging behaviour was identified during chart review based on documented behavioural descriptions (e.g., anger, aggression, irritability, agitation, and confusion). The tool also captured the use of formal assessment tools recorded (e.g., delirium and cognitive screening instruments, behaviour charts, psychological assessments, capacity assessment Deprivation of Liberty Safeguards, or enhanced care risk assessments).

Data Analysis

Descriptive statistics were used to summarise data for all patients and for subgroups based on the documented presence or absence of challenging behaviour. Frequencies, percentages, means, medians, and ranges were calculated where relevant. Subgroups are presented based on the documented presence or absence of challenging behaviour, across demographic, clinical, and discharge-related variables. For each variable, reporting was based on clinical record documentation. Where information was missing, this is reflected in table denominators.

Ethical Considerations

This project was registered and approved as a clinical audit within the NHS Trust. Under NHS governance procedures, NHS Research Ethics Committee review is not required for registered clinical audits. Analysis and publication of anonymised audit data are permitted. Patient records were anonymised prior to analysis, and no direct patient contact occurred.

## 3. Results

This study examined 48 patients following a stroke, with challenging behaviour documented in 13 patients during their stay (see Table 1). Ages were comparable in both groups, and women were more represented among those with challenging behaviour (8/13). Around half of those who developed challenging behaviour had been living independently (7/13), with nearly a third in residential or nursing care (4/13). Most patients who developed challenging behaviour had experienced an ischaemic stroke (11/13), with only two cases following intracerebral haemorrhage. Patients with challenging behaviour were also more often categorised as having moderate stroke severity on admission (6/13), and median length of stay was longer for those with challenging behaviour (19 days) compared to those without (7 days).

Only 3 of the 13 patients in whom challenging behaviour was documented had a recorded history of confusion or challenging behaviour before their stroke. Dementia was documented in three patients, and previous stroke in only one patient. Most had other pre-existing health conditions (12/13). Clinical factors were noted in 9 of the 13 patients at the time their behaviour was observed, most commonly inattention/neglect (n = 5) and infection (n = 4), and several patients had other acute contributing factors such as frailty, communication problems, or impulsivity (n = 5). See Table 2 for history and clinical factors.

When challenging behaviour was documented, challenges to care delivery were documented in almost half (n = 6) of these patients, predominantly through refusal of treatment (n = 4). Challenging behaviour occasionally disrupted the care of other patients (n = 2) and additional professional input was required for over half of the patients with challenging behaviour (n = 7), including nursing staff, and specialist clinical and multidisciplinary teams. Management strategies were documented for all patients with challenging behaviour and included a range of clinical, safeguarding, and supportive approaches, most commonly one-to-one care, and deprivation of liberty standards (both n = 6). See Table 3 for documented impact on care and management.

Post-discharge destinations for patients in whom challenging behaviour was documented were varied (See Table 4). Of the 11 with recorded outcomes, 6 returned to their own homes independently or with family assistance, with 4 being discharged to a nursing home. Discharge was delayed for 4 of the 13 patients in whom challenging behaviour was documented, due to the need for either an increased package of care (n = 2) or a specialist discharge destination (n = 2).

Documentation of behavioural presentation showed that challenging behaviour was most often described as confusion or agitation, and reports were usually made by nursing or therapy staff. Where follow-up assessments were recorded, these commonly related to capacity, deprivation of liberty, or risk assessments, while formal cognitive screening was only recorded for one patient.

## 4. Discussion

This study examined challenging behaviour following acute stroke in relation to patient characteristics, management, and impact on care. Challenging behaviour was recorded in just over a quarter of patients admitted with stroke, with caution that this reflects clinical documentation and perception rather than prevalence per se. Those in whom challenging behaviour was documented were typically older adults, more often women, admitted with moderate strokes and a range of co-existing health conditions. Consequently, challenging behaviours in stroke were documented alongside clinical complexity rather than a distinct or predictable patient profile. Inattention/neglect and infection were commonly recorded clinical factors documented alongside challenging behaviour. When documented, challenges to care delivery were documented in around half of patients, most notably treatment refusal and the need for additional nursing or multidisciplinary input. Admissions were also longer for these patients, occurring in the context of management and discharge planning complexity. Management approaches varied and challenging behaviour was occasionally documented alongside discharge delays for specialist care planning or placement arrangements. This variability suggests that the role of early specialist multidisciplinary involvement in managing care complexity could be explored in future research. Behaviours were most often recorded by nursing staff and categorised predominantly as confusion or agitation. Despite this, formal cognitive screening was infrequently documented, but it was unclear whether this reflected actual assessment or merely documentation practices. Understanding staff use and documentation of structured screening and assessment tools would help clarify this finding.

The profile of patients with documented challenging behaviour in this study (i.e., older adults, mostly women, with moderate stroke severity and multiple comorbidities) both contrasts with and complements previous research. Earlier studies have linked post-stroke aggression to younger age, a history of depression, speech difficulties, and poor cognitive or motor function [24,25,26,27]. In contrast, Lau et al. [10] found no association between post-stroke anger and age or gender. However, in line with current findings, previous health conditions, such as diabetes, concurrent depression, and stroke severity have been linked to post-stroke aggression and agitation [10,20]. Alignment with some studies and divergence from others may reflect the fact that the literature is yet to converge on a consistent understanding of post-stroke challenging behaviour, including how behaviours are defined and labelled.

Inattention, neglect, and infection were commonly documented among patients with challenging behaviour in this study. Santos et al. [17] noted neglect among patients with intense anger, while Choi-Kwon and Kim [3] described post-stroke anger as a manifestation of delirium, involving disturbances in attention and cognition. Infection may also contribute to behavioural disturbance by precipitating delirium, a link supported in stroke populations [28] and more broadly in older adults [29]. These findings are consistent with our study, where confusion and agitation were frequently documented. However, formal cognitive or delirium assessments were rarely recorded, limiting insight into behavioural assessment practices. In the absence of formal screening to identify potential cognitive or physiological contributors, pathways to specialist input are less clear, increasing reliance on non-specialist management within routine care.

Documented challenges to care included treatment refusal, increased multidisciplinary input, and longer admissions, reflecting tangible service pressures during acute stroke care. Consequently, processes that support timely identification and escalation of care could be explored in future research. This reflects earlier work from Remer-Osborn [30], who described behavioural disturbances as contributing to caregiver distress and communication breakdown. While caregiver experience was beyond the scope of this audit, its prominence in the literature highlights the importance of future work that considers emotional wellbeing and organisational support mechanisms among stroke caregivers. The absence of standardised tools for assessing post-stroke behavioural symptoms, as noted by Choi-Kwon and Kim [3], and the under-recognition of these behaviours in stroke care, suggest the value in exploring improved screening and management protocols. This need is also supported by van Heugten and Wilson [21] and the National Clinical Guideline for Stroke [31], which advocate for early identification and tailored neuropsychological support to improve rehabilitation outcomes. In summary, the literature and current findings suggest that challenges in care delivery documented in relation to challenging behaviour may be well recognised but remain difficult to address consistently.

These findings offer insight into how challenging behaviour is recognised, documented, and managed within routine stroke services, informing consideration of broader implications for service delivery and future research.

Implications and future directions

This study provides a descriptive snapshot of challenging behaviour presentation and management within a single NHS Trust. While the findings cannot be generalised beyond this setting, they identify patterns that could be explored further locally and nationally. Challenging behaviours in clinically complex situations, as reflected in documentation alongside moderate stroke severity, comorbidities, and additional care needs observed here, may benefit from multidisciplinary and multi-specialty assessment to ensure medical and behavioural contributors are appropriately addressed. The infrequent documentation of formal cognitive or delirium screening found in this study suggests inconsistency in recorded behavioural assessment practices. However, this may not reflect actual clinical practice, highlighting scope in exploring screening and its documentation to support clinical response further. If similar patterns are found in larger studies, wider specialty-specific protocols could be explored in a national context. The predominance of ‘confusion’ and ‘agitation’ as descriptors found here highlights the challenge of defining and categorising behavioural change after stroke. This has implications for care, as distinguishing cognitive states from behaviours informs appropriate assessment and response. Further work could explore how challenging behaviour is operationalised in stroke care to support recognition, documentation, and management. The observed difference in length of stay indicates an area for further investigation in larger cohorts, with statistical testing to characterise these patterns, and to establish whether similar patterns exist in other settings. These descriptive findings provide a preliminary basis for larger-scale, multi-site research that may inform practice recommendations.

Strengths and limitations

Consecutive stroke admissions during the study period were reviewed, reducing the risk of selection bias. The inclusion of two units representing different points in the stroke pathway provided some contextual breadth in how behaviours were recorded and managed. The study also establishes a foundation for future, larger-scale evaluation of behavioural presentation and management across stroke services. The study was conducted within a defined time period associated with a research internship, which limited the duration of data collection to that period. Consequently, findings may not reflect patterns across different time periods. Within this time-limited scope, the data extraction tool was not pilot tested, and data extraction consistency was not formally assessed. As the study was retrospective, it relied entirely on existing clinical notes. The information available depended on what had been recorded at the time, which may have reflected clinician variability, and interpretation by a single reviewer may have introduced subjectivity. Similarly, with the retrospective design, we were unable to clarify ambiguities in the clinical record or determine whether the clinical notes fully reflected actual care and management. Challenging behaviour was not defined using a standardised operational definition, and identification relied on terminology used in routine clinical documentation. Accordingly, misclassification related to documentation is a potential source of bias in this study. Finally, the small sample from a single organisation and geographical area may not reflect settings elsewhere.

## 5. Conclusions

Challenging behaviour was documented in just over a quarter of stroke admissions, most often described as confusion or agitation, and was commonly documented alongside inattention or infection. When documented, it was done so alongside challenges in care delivery, sometimes through treatment refusal, and often alongside additional multidisciplinary input. Length of stay was longer for these patients, and discharge was sometimes delayed. Formal cognitive or delirium screening was rarely recorded. These findings provide a descriptive account of the way challenging behaviour was documented in stroke care at a local level. They provide baseline descriptive data to support a broader national exploration of how such behaviours are recognised, understood, and managed in clinical practice.

## Figures and Tables

**Table 1:** Demographic and clinical characteristics by documented presence of challenging behaviour.

Variable	Challenging Behaviour (n = 13)	No Challenging Behaviour (n = 35)
Age		
Mean (SD)	73.15 (16.16)	72.54 (11.59)
Range in years	44–91	45–89
Gender		
Female	8 (61.5%)	15 (42.9%)
Male	5 (38.5%)	20 (57.1%)
Pre-admission care		
Independent/No formal care	7 (53.8%)	28 (80%)
Care by family	2 (15.4%)	4 (11.4%)
Professional care	0 (0%)	2 (5.7%)
24 h care	4 (30.8%)	1 (2.9%)
Stroke type		
Ischaemic	11 (84.6%)	29 (82.9%)
Intracerebral Haemorrhage	2 (15.4%)	6 (17.1%)
Stroke severity (NIHSS category) *		
0 or normal	2 (15.4%)	1 (3.1%)
1–4 minor stroke	2 (15.4%)	16 (50.0%)
5–15 moderate stroke	6 (46.1%)	8 (25%)
16–20 moderate–severe stroke	2 (15.4%)	3 (9.4%)
21–42 severe stroke	1 (7.7%)	4 (12.5%)
Length of stay, days		
Median (Q1–Q3, range) **	19 (13–27, 3–37)	7 (3–40, 1–375)

* Recorded for n = 45 only. ** Data presented as median (Q1–Q3, range) due to skewed distribution and presence of outliers.

**Table 2:** Clinical history and clinical factors by documented presence of challenging behaviour.

Variable	Challenging Behaviour (n = 13)	No Challenging Behaviour (n = 35)
Pre-stroke Hx of confusion/challenging behaviour		
Yes	3 (23.1%)	3 (8.6%)
No	10 (76.9%)	32 (91.4%)
	Challenging Behaviour (n = 13)	No Challenging Behaviour (n = 34 *)
Pre-stroke medical history **		
Previous stroke	1 (7.7%)	8 (22.9%)
Dementia	3 (23.1%)	1 (2.9%)
Confusion/Agitation	2 (15.4%)	1 (2.9%)
Psychiatric illness	1 (7.7%)	2 (5.7%)
Anger/Aggression	0 (0%)	1 (2.9%)
Other	12 (92.3%) ***	22 (62.9%)
Clinical factors noted for challenging behaviour **		
≥1 noted	9 (69.2%)	14 (40.0%)
Inattention/Neglect	5 (38.5%)	7 (20.0%)
Infection	4 (30.8%)	5 (14.3%)
Pain	1 (7.7%)	0 (0%)
Other	5 (38.5%) ****	8 (22.9%)

* Pre-stroke medical history and clinical factors were not documented for one patient. ** Patients could be recorded in more than one category; therefore, percentages do not sum to 100%. *** Cardiovascular, metabolic, mental health, sensory, and frailty-related conditions. **** Acutely ill; communication, speech impairment; language impairment; severe frailty, dense weakness on right; very impulsive, lacking awareness and insight.

**Table 3:** Documented impact on care and subsequent management strategies.

Variable	Challenging Behaviour (n = 13)
Impact on patient care (6, 46.2%) *	
Refused food/drink	1 (7.7%)
Refused treatment	4 (30.8%)
Not sleeping	2 (15.4%)
Impact on other patients *	
Disrupted care	2 (15.4%)
Additional staff/professional input (7, 53.8%) *	
Nursing staff (nurse/HCA)	3 (23.1%)
Specialist clinical teams **	3 (23.1%)
Broader hospital/MDT ***	2 (15.4%)
Management strategy documented (13, 100%) *	
Medication review	5 (38.5%)
Deprivation of Liberty Safeguards	6 (46.2%)
Psychiatrist involvement	1 (7.7%)
One-to-one care	6 (46.2%)
Family involvement	3 (23.1%)
Distraction techniques	1 (7.7%)
Other ****	7 (53.8%)

* Percentages are calculated from all patients with challenging behaviour (n = 13); totals may exceed the base sample as some patients were recorded in more than one category. ** Psychologist, palliative care, nutritionist. *** Hospital at Night team, MDT discussions, medical staff (doctor/pathology). **** Monitoring by nursing staff, mitts/bed rails, specialist equipment, sensory items from home, engagement/distraction by staff, and orientation work with occupational therapy.

**Table 4:** Discharge outcomes by documented presence of challenging behaviour.

Variable	Challenging Behaviour (n = 13)	No Challenging Behaviour (n = 35)
Post-discharge living destination *		
Own home (Independent or family-assisted)	6 (54.5%)	23 (76.7%)
With family	1 (9.1%)	3 (10%)
Residential home	0 (0%)	1 (3.3%)
Nursing home	4 (36.4%)	3 (10%)
Discharge delayed due to challenging behaviour		
Yes	4 (30.8%)	n/a
No	9 (69.2%)	
Reason for delay (if yes) (n = 4)		
Need for an increased package of care	2 (50%)	n/a
Need for a specialist discharge destination	2 (50%)	

* N = 41; n = 7 had no post-discharge destination recorded (CB n = 2, No CB n = 5). Percentages are calculated from valid responses only (CB n = 11, No CB n = 30).

## Data Availability

The aggregated data supporting the findings of this study are presented within the article. Individual patient-level data cannot be shared due to patient privacy and confidentiality considerations.

## Supplementary Materials

The following supporting information can be downloaded at: https://www.mdpi.com/article/10.3390/nursrep16020053/s1, File S1. Revised Standards for QUality Improvement Reporting Excellence (SQUIRE 2.0) publication guidelines.

## Public Involvement Statement

There is no public involvement in any aspect of this research.

## Guidelines and Standards Statement

This manuscript was drafted in accordance with the SQUIRE 2.0 (Standards for Quality Improvement Reporting Excellence) guidelines for reporting quality improvement and clinical audit studies [23].

## References

[1] NHS England (2024). NHS Launches Major New Stroke Campaign as Thousands Delay Calling 999 by Nearly 90 Minutes. https://www.england.nhs.uk/2024/11/nhs-launches-major-new-stroke-campaign-as-thousands-delay-calling-999-by-nearly-90-minutes/.

[2] Kusec A., Milosevich E., Williams O.A., Chiu E.G., Watson P., Carrick C., Drozdowska B.A., Dillon A., Jennings T., Anderson B. (2023). Long-term psychological outcomes following stroke: The OX-CHRONIC study. BMC Neurol..

[3] Choi-Kwon S., Kim J.S. (2022). Anger, a result and cause of stroke: A narrative review. J. Stroke.

[4] Fabricius J., Andersen A.B., Lindegård Munk G., Kaae Kristensen H. (2024). Confused about Rehabilitation? Multi-Faceted Approaches for Brain Injured Patients in a Confusional State. Hospitals.

[5] Brunero S., Lamont S., Foster K., Marks P., O’Brien A., Hurley J. (2024). Safety in care, safety at work. Mental Health Nursing: Theory and Practice for Clinical Settings.

[6] Lamont S., Brunero S., Roberts L., Haines D. (2022). Managing challenging behaviours, aggression and violence. Mental Health and Mental Illness in a Pre-Hospital Setting.

[7] Kneebone I.I., Lincoln N.B. (2012). Psychological problems after stroke and their management: State of knowledge. Neurosci. Med..

[8] Ferro J.M., Santos A.C. (2020). Emotions after stroke: A narrative update. Int. J. Stroke.

[9] Kim J.S. (2024). Neuropsychiatric Manifestations in Neurological Diseases.

[10] Lau C.G., Tang W.K., Liu X.X., Liang H.J., Liang Y., Wong A., Mok V., Ungvari G.S., Wong K.S., Kim J.S. (2017). Poststroke agitation and aggression and social quality of life: A case control study. Top. Stroke Rehabil..

[11] Pouwels C., Spauwen P., Van Heugten C., Botteram R., Winkens I., Ponds R. (2024). Dealing with patients with behavioural problems after acquired brain injury: Nurses’ experiences with the antecedent behaviour consequence method. J. Long Term Care.

[12] Winkens I., van Heugten C., Pouwels C., Schrijnemaekers A.-C., Botteram R., Ponds R. (2019). Effects of a behaviour management technique for nursing staff on behavioural problems after acquired brain injury. Neuropsychol. Rehabil..

[13] Lapchak P.A. (2015). Neuronal dysregulation in stroke-associated pseudobulbar affect (PBA): Diagnostic scales and current treatment options. J. Neurol. Neurophysiol..

[14] Shi Q., Presutti R., Selchen D., Saposnik G. (2012). Delirium in acute stroke: A systematic review and meta-analysis. Stroke.

[15] Patel K., Holland E.-J., Watkins C.L., Bowen A., Read J., Thomas S., Roberts T., Lightbody C.E. (2025). Exploring staff perspectives on implementing an intervention package for post-stroke psychological support: A qualitative study. Psychol. Int..

[16] Griffiths M., Kontou E., Ford C. (2023). Psychological support after stroke: Unmet needs and workforce requirements of clinical neuropsychological provision for optimal rehabilitation outcomes. Br. J. Hosp. Med..

[17] Santos C., Caeiro L., Ferro J., Albuquerque R., Figueira M.L. (2006). Anger, hostility and aggression in the first days of acute stroke. Eur. J. Neurol..

[18] Santos C.O., Caeiro L., Ferro J.M., Figueira M.L. (2011). Mania and Stroke: A systematic review. Cerebrovasc. Dis..

[19] Kumral E., Çetin F.E., Özdemir H.N., Çelikay H., Özkan S. (2024). Post-stroke aggressive behavior in patients with first-ever ischemic stroke: Underlying clinical and imaging factors. Acta Neurol. Belg..

[20] Choi-Kwon S., Han K., Cho K.H., Choi S., Suh M., Nah H.W., Kim J. (2013). Factors associated with post-stroke anger proneness in ischaemic stroke patients. Eur. J. Neurol..

[21] van Heugten C.M., Wilson B.A., Platz T. (2021). Cognition, emotion and fatigue post-stroke. Clinical Pathways in Stroke Rehabilitation: Evidence-Based Clinical Practice Recommendations.

[22] Kim J.S. (2016). Post-stroke mood and emotional disturbances: Pharmacological therapy based on mechanisms. J. Stroke.

[23] Ogrinc G., Davies L., Goodman D., Batalden P., Davidoff F., Stevens D. (2016). SQUIRE 2.0 (Standards for QUality Improvement Reporting Excellence): Revised publication guidelines from a detailed consensus process. BMJ Qual. Saf..

[24] Aybek S., Carota A., Ghika-Schmid F., Berney A., Van Melle G., Guex P., Bogousslavsky J. (2005). Emotional behavior in acute stroke: The Lausanne emotion in stroke study. Cogn. Behav. Neurol..

[25] Chan K.-L., Campayo A., Moser D.J., Arndt S., Robinson R.G. (2006). Aggressive behavior in patients with stroke: Association with psychopathology and results of antidepressant treatment on aggression. Arch. Phys. Med. Rehabil..

[26] Paradiso S., Robinson R.G., Arndt S. (1996). Self-reported aggressive behavior in patients with stroke. J. Nerv. Ment. Dis..

[27] Kim J.S., Choi S., Kwon S.U., Seo Y. (2002). Inability to control anger or aggression after stroke. Neurology.

[28] Kowalska K., Droś J., Mazurek M., Pasińska P., Gorzkowska A., Klimkowicz-Mrowiec A. (2020). Delirium post-stroke: Short- and long-term effect on depression, anxiety, apathy and aggression. J. Clin. Med..

[29] Krinitski D., Kasina R., Kloeppel S., Lenouvel E. (2021). Associations of delirium with urinary tract infections and asymptomatic bacteriuria in adults aged 65 and older: A systematic review and meta-analysis. J. Am. Geriatr. Soc..

[30] Remer-Osborn J. (1998). Psychological, behavioral, and environmental influences on post-stroke recovery. Top. Stroke Rehabil..

[31] National Institute for Health and Care Excellence (2023). Stroke Rehabilitation in Adults, NICE. https://www.nice.org.uk/guidance/ng236.

